# Antipredatory Function of Head Shape for Vipers and Their Mimics

**DOI:** 10.1371/journal.pone.0022272

**Published:** 2011-07-27

**Authors:** Janne K. Valkonen, Ossi Nokelainen, Johanna Mappes

**Affiliations:** Centre of Excellence in Evolutionary Research, Department of Biological and Environmental Science, University of Jyväskylä, Jyväskylä, Finland; Michigan State University, United States

## Abstract

Most research into the adaptive significance of warning signals has focused on the colouration and patterns of prey animals. However, behaviour, odour and body shape can also have signal functions and thereby reduce predators' willingness to attack defended prey. European vipers all have a distinctive triangular head shape; and they are all venomous. Several non-venomous snakes, including the subfamily Natricinae, commonly flatten their heads (also known as head triangulation) when disturbed. The adaptive significance of this potential behavioural mimicry has never been investigated.

We experimentally tested if the triangular head shape typical of vipers offers protection against predation. We compared the predation pressure of free-ranging predators on artificial snakes with triangular-shaped heads against the pressure on replicas with narrow heads. Snakes of both head types had either zigzag patterned bodies, typical of European vipers, or plain (patternless) bodies. Plain snakes with narrower Colubrid-like heads suffered significantly higher predation by raptors than snakes with triangular-shaped heads. Head shape did not, however, have an additive effect on survival in zigzag-patterned snakes, suggesting that species which differ from vipers in colouration and pattern would benefit most from behavioural mimicry. Our results demonstrate that the triangular head shape typical of vipers can act as a warning signal to predators. We suggest that head-shape mimicry may be a more common phenomenon among more diverse taxa than is currently recognised.

## Introduction

Research into warning signals and defensive mimicry has largely concentrated on colouration and colour patterns in prey animals. Body shape, odour, behaviour or a combination of these features may also act as warning signals and can therefore be mimicked by other species [1]–[5]. Myrmecomorphy, among the group of spiders that mimic ants, is a famous example of behavioural mimicry. Myrmecomorphs often reinforce their morphological resemblance to ants through mimicry of ant leg movements and zigzag walking [6]–[7]. Behavioural mimicry combined with colouration has also been recorded among cephalopods: long-armed octopus species inhabiting Indonesian waters mimic venomous sea snakes [8]. When disturbed, these animals alter their colouration to present black and white bands, and then adopt a specific posture. Norman et al. [8] have reported cases where six of the octopus arms were hidden in the burrow while the remaining two arms were held straight, away from the burrow, imitating a local sea snake. Perhaps the most famous example of behavioural mimicry was described by Henry Bates in 1862 who observed that some butterfly larvae seem to mimic snakes. When the late instar larvae of swallowtail butterflies (*Papilo sp.*) and hawk moths (Sphingidae) are threatened by a predator, they mimic small tree vipers by hiding their heads and inflating the thorax or abdomen [1], [9]–[10].

**Figure 1:**
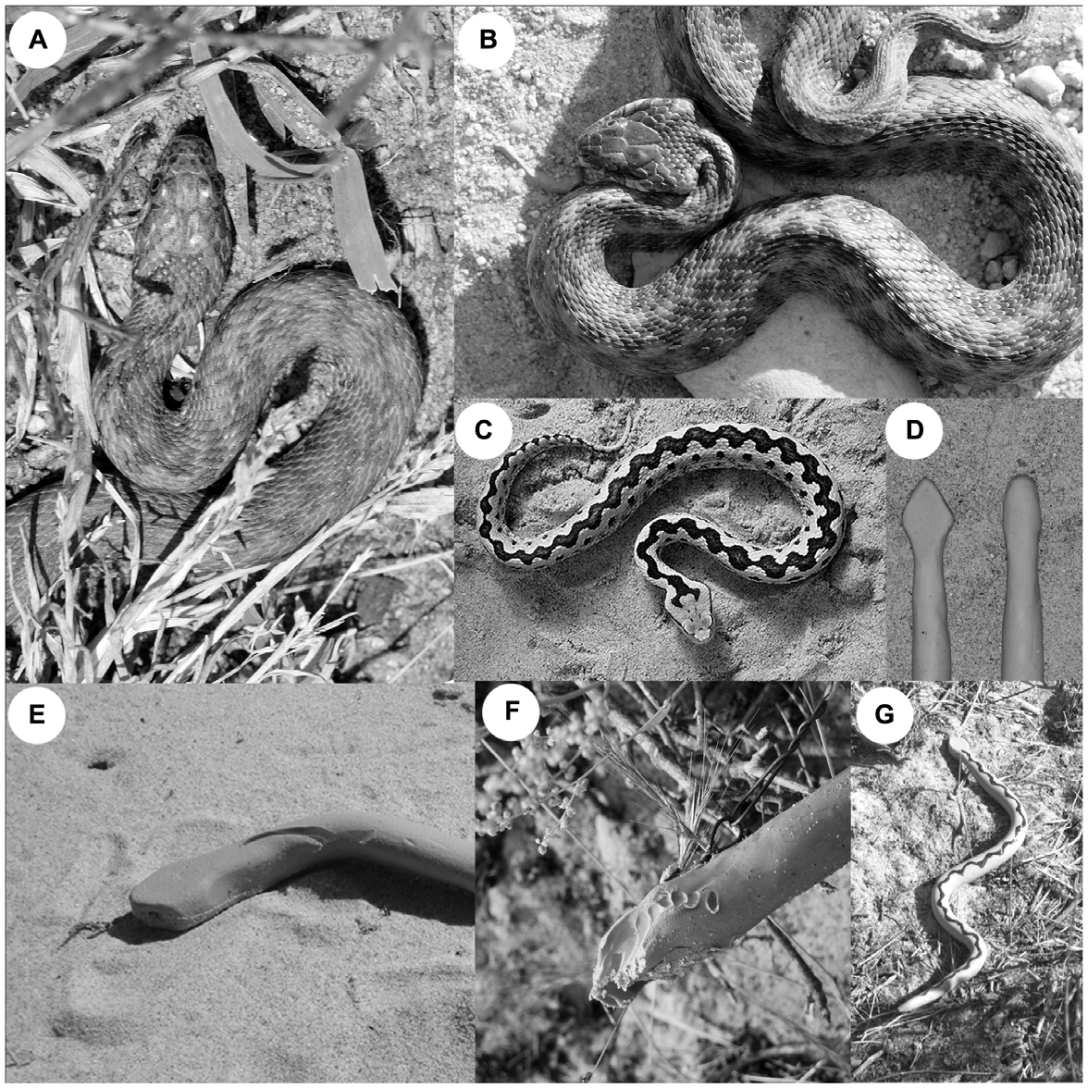
Head shapes of viper and viperine snakes. *Natrix maura* has a narrow colubrid-like head shape (a). When disturbed, they flatten their heads making the head more triangular in shape (b). *Vipera latastei gaditana* exhibit the typical triangular head shape of European vipers (c). Triangular, viper-like and narrow, colubrid-like head shapes of plasticine models (d). Attacks of raptors (e) and mammals (f) can be separated from imprints left during predation events. A dorsal zigzag pattern was painted on half of the snake replicas (g).

A triangular head shape and dorsal zigzag pattern are common features among venomous European vipers (genus *Vipera*) [11] (Fig. 1c). However, non-venomous snakes in the family Colubridae [12] often have a narrower head shape. Some, such as viperine snakes (*Natrix maura*) [13]–[14] (Fig. 1a), grass snakes (*Natrix natrix*) and smooth snakes (*Coronella austriaca*) (personal observation) flatten their heads (head triangulation) when disturbed (Fig. 1b). Viperine snakes exhibit a dorsal zigzag pattern that resembles vipers' zigzag pattern whereas grass snakes and smooth snakes commonly do not [15]. Viperine snakes have been observed excreting a strong-smelling liquid from their cloacal glands when disturbed, which may be unpleasant to predators [15] and, thus, this species could be considered a Müllerian [2] or quasi-Batesian viper mimic [5], [16]. Head triangulation alone or combined with a viper-like dorsal zigzag pattern may improve non-venomous snakes' resemblance to vipers [15]. If several groups of predators avoid vipers based on their triangular head shape, a range of species could benefit from mimicry of this head shape.

The significance of head triangulation and its advantages for mimics have not been empirically tested. To experimentally test if a triangular head shape, and the head triangulation in particularly, acts as a warning signal, we compared predation caused by free-ranging predators on snake replicas with triangular (viper-like) and narrow (colubrid-like) heads. To differentiate the effect of the triangular head shape from the overall appearance of European vipers we used snake replicas with and without the characteristic zigzag pattern typical of European vipers (Fig. 1e,g).

## Materials and Methods

Following previously employed methods [17]–[23], we used artificial snakes made of non-toxic grey plasticine (Caran D'Ache, Modela Noir, 0259.005). We painted the characteristic zigzag pattern on half of them using black paint (Bebeo acryl colour 374611 & Perinnemaali 5511-05) (Fig. 1. g). A 50∶50 mixture of two types of paint were used to achieve a matte-black finish. To test if vipers' triangular head shape acts as a warning signal, four different kinds of artificial snakes were used: 1) zigzag-patterned snakes with triangular (viper type) heads and 2) with narrow (colubrid type) heads and 3) plain (grey) snakes with triangular heads and 4) with narrow heads (Fig. 1d,e,g). The use of plain snakes was crucial to separate the effect of the head shape from the overall appearance of vipers. The length and diameter of artificial models were identical in all treatments and in correspondence to the size of a sub adult/adult viper (*Vipera latastei gaditana*) [11], [15]. This was confirmed by measuring 82 randomly-chosen clay models (range = 36–45 cm, mean = 41.8 cm, s.e. = 0.2 cm). The models did not differ in length between treatments (*F*_3.78_ = 1.10, *p* = 0.355). The average width of the triangular heads was 27.8 mm (s.e. = 0.5 mm, *n* = 12) and of narrow heads 18.3 mm (s.e. = 0.3 mm, *n* = 13) corresponding to the natural variation of both viper and viperine snake head width (Fig. 1d).

The experiment was conducted in Coto Doñana National Park, southern Spain, between 5 and 17 May 2009 and 28 April and 10 May 2010. Six trials (transects) were conducted in 2009 and thirteen in 2010. Trials were conducted in 17 different locations 0.5–38.9 km apart (mean 9.7 km). We used five to ten replicas of each type (4 types) in each transect (595 snake replicas in total). The replicas were placed on the natural background in random order at approximately 15 metre (15 paces) intervals following features of the terrain. All model types were equally represented within transects. However, one zigzag patterned model with a triangular head was accidentally omitted from one transect (nine replicas instead of ten). Between one and four trials were conducted simultaneously and with a minimum distance of 2.5 km between them. Snake replicas were tied to bushes with iron wire to prevent predators taking them during an attack. The replicas were sprayed with insect repellent (Autan® by Johnson) making them distasteful to deter mammals (mainly foxes and boars) from eating them. In a previous experiment [23], we observed foxes following our tracks along transect lines and systematically biting every snake replica, destroying all traceable evidence of attacks by other predators. The use of insect repellent significantly reduced this indiscriminate predation.

In 2009, snake replicas were first checked after approximately 12 hours and then again after approximately 24 hours. After approximately 48 hours in the field, they were checked a final time and then removed. In 2010, snake replicas were left in the field for a longer period to accumulate more attacks. The prey items were checked every 24, 48 and 72 hours and the number of attacks on each item recorded. All the attacked snake replicas were repaired after each inspection so that the probability of models being attacked remained constant. Attacks by raptors and mammals, e.g. foxes, were recorded separately. Raptor attacks (Fig. 1e) can be separated from those caused by corvids, gulls or mammals (Fig. 1f) by the imprints that result from the attacks. While raptors use their talons during an attack, gulls and corvids tend to approach the prey from the ground and use their beaks. The footprints of the diverse predators were easily detectable in the soft sandy soil of the experimental areas. No corvid or gull attacks were observed during the experiment.

Raptors can fly long distances in a short period of time making it likely that an individual raptor saw snake replicas in several transects, violating the independence of transect lines. Therefore data from all trials was treated as one independent sample. The Pearson Chi-squared test of independence was used to test differences in the number of attacks on each of the four snake types. There was no significant main effect or interaction between years and attack rates (neither of mammals nor of raptors) among treatments (Logit model, all *Z* values≤±1.618, and *p* values≥0.106) and thus we pooled the data from both years.

## Results

**Figure 2:**
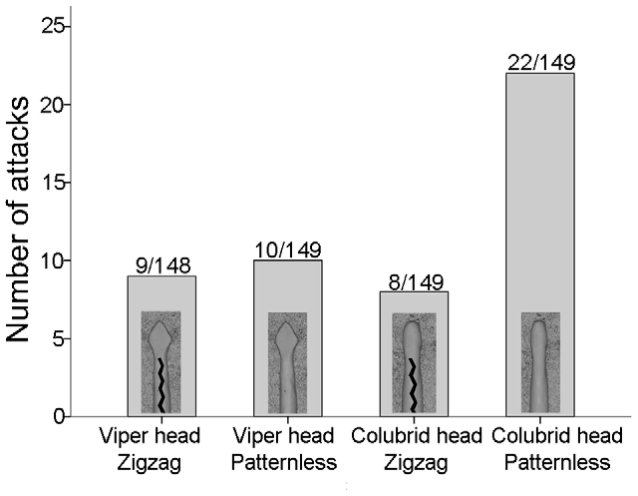
The number of attacks on snake replicas. The observed number of raptor attacks on different types of snake replicas. Numbers at the top of each bar represent the total number of observed raptor attacks on each type of snake replica in relation to the total number of replicas of that type used in the experiment.

There was a significant difference in the number of raptor attacks among treatments (χ^2^ = 11.393, *df* = 3, *N* = 595, *p* = 0.010). Plain snakes with narrow heads were attacked significantly more often by raptors than were plain snakes with triangular heads (*χ^2^* = 5.04, *df* = 1, *p* = 0.025) (Fig. 2). There was no difference in the number of raptor attacks on patterned, triangular-headed snake replicas and on patterned replicas with narrow heads (*χ^2^* = 0.07, *df* = 1, *p* = 0.792) (Fig. 2). When we pooled attack data on snake replicas based on their patterns, plain snakes were attacked by raptors significantly more frequently than were zigzag patterned replicas (χ^2^ = 6.49, *df* = 1, *p* = 0.011). In total, 8.2% of the 595 snake replicas were attacked by raptors and 18.5% by mammalian predators. Attacks by mammalian predators did not differ between treatments (χ^2^ = 5.10, *df* = 3, *p* = 0.165).

## Discussion

We have shown that the triangular head shape of vipers is recognized and avoided by raptors and does, therefore, act as a warning signal. Plain, triangular-headed snake replicas suffered significantly fewer raptor attacks than suffered by plain replicas with narrow heads. Moreover, plain snake replicas were attacked more often overall than were patterned replicas. However, a triangular head shape did not have any antipredator benefit when presented together with a zigzag-patterned body, suggesting that pattern and colouration together are a sufficient warning signal. Many colubrid species that display head triangulation notably do not mimic the body pattern of vipers. Our results also suggest that behavioural mimicry (head triangulation) can, in particular, significantly increase the survival of species that do not mimic the body pattern of vipers.

Although raptors recognized head shape as a signal, we did not find any evidence that mammalian predators avoided the triangular head shape or zigzag pattern of the snake replicas. Using plasticine prey items might not be a suitable method to study mammalian predation, as mammals use olfactory cues rather than visual cues during hunting. The odour of plasticine is distinctive and that can influence predators' behaviour [24]. Previously, we observed foxes following our tracks along transect lines and systematically biting and eating every snake replica in their path [23]. These observations suggest that mammalian predators (e.g. foxes) do not consider plasticine prey items as real living (or dead) snakes. It would be interesting to study the efficacy of head-flattening behaviour against mammalian predators using replicas with various olfactory cues.

Vipers (family Viperidae) are venomous, widely distributed and tend to be avoided by predators [21]–[23], making them good models for several groups of animals. We suggest that the triangular head shape of venomous snakes is mimicked by prey animals more broadly than was previously thought. Indeed, potential head shape mimicry has independently evolved several times among snakes. Horizontal head display is known to exist among 13 Old World and 9 New World genera of the families Colubridae, Elapidae, and Viperidae [25]. Snakes commonly flatten or inflate their bodies as defensive behaviour to make themselves appear bigger than they are [26]. Enlargement may occur over the whole body or it may be concentrated in a particular area, such as the neck or head [26]. While head triangulation in snakes is often combined with body flattening/inflating behaviour, it also occurs among species that do not exhibit body-flattening behaviour. Smooth snakes (*Coronella austriaca*), for example, flatten (triangulate) their heads when disturbed but do not flatten their bodies (personal observation). Given the taxonomic and geographic diversity of these genera, much of the similarity in defensive behaviour must be due to convergence [25], which we suggest has been driven by defensive mimicry. Further support for these behavioural observations comes from Young et al. [14] who describe the mechanical basis of head triangulation in distantly-related colubrid snakes. They found that the morphological mechanisms required to triangulate the head differ greatly between the genus *Hetrodon* and *Dasypeltis*.

In addition to snakes, the larvae of several moth and butterfly species enhance their resemblance to tree vipers by concealing their heads and inflating their thorax or abdomen to express a false triangular-shaped head [1], [9]–[10]. Hawkmoth (*Leucorampha sp.*) caterpillars also “strike” the objects that threaten them making the behavioural mimicry even more accurate [26]. Although the adaptive significance of this behaviour against predators has never been tested experimentally, it is reasonable to assume that the triangular head shape is effective against predators as it is known to be against humans. Henry Bates described the power of this mimicry in 1862; “The most extraordinary instance of imitation I ever met with was that of a very large Caterpillar, which stretched from amidst the foliage of a tree which I was one day examining, and startled me by its resemblance to a small Snake. … I carried off the Caterpillar, and alarmed every one in the village where I was then living, to whom I showed it.”[1]. The fascinating anecdotal evidence combined with intriguing research data argue the need for a survey other animals to discover if head-shape mimicry can be found elsewhere in the animal kingdom.
